# MicroRNA-384 Inhibits the Progression of Papillary Thyroid Cancer by Targeting PRKACB

**DOI:** 10.1155/2020/4983420

**Published:** 2020-01-08

**Authors:** Yongxia Wang, Beixi Wang, Hong Zhou, Xiangnan Zhang, Xinlai Qian, Jing Cui

**Affiliations:** ^1^Department of Pathology, School of Basic Medical Sciences, Xinxiang Medical University, Xinxiang 453003, Henan, China; ^2^Department of Pathology, Third Affiliated Hospital of Xinxiang Medical University, Xinxiang 453003, Henan, China; ^3^Henan Provincial Key Laboratory of Molecular Tumor Pathology, Henan, Xinxiang, China

## Abstract

**Background:**

Growing evidence shows that dysregulation of miRNAs plays a significant role in papillary thyroid cancer (PTC) tumorigenesis and development. The abnormal expression of miR-384 has been acknowledged in the proliferation or metastasis of some cancers. However, the function and the underlying mechanism of miR-384 in PTC progression remain largely unknown.

**Methods:**

Real-time PCR was conducted to detect miR-384 expression in 58 cases of PTC and their adjacent noncancerous tissues. MTT, soft agar assay Transwell assay, and wound-healing assay were carried out to explore the biological function of miR-384 in PTC cell lines of BCPAP and K1. Bioinformatics analysis, dual-luciferase reporter assay, western blot, and functional complementation analysis were conducted to explore the target gene of miR-384. Moreover, Spearman's correlation analysis was conducted to reveal the correlation between miR-384 and PRKACB mRNA in PTC.

**Results:**

The expression of miR-384 decreased obviously in PTC, especially in the tumors with lymph node metastasis or larger tumor size. The ectopic upregulation of miR-384 significantly suppressed PTC progression, and the inhibition of miR-384 had the opposite effects. Moreover, PRKACB gene was confirmed as the target of miR-384.

**Conclusion:**

The study suggests that miR-384 serves as a tumor suppressor in PTC progression by directly targeting the 3′-UTR of PRKACB gene.

## 1. Introduction

Papillary thyroid cancer (PTC) is the most common subtype of thyroid malignancy with approximately 77% diagnosed in women [1]. In addition, the incidence of PTC has been increasing in the past few years [2]. And many factors have been recognized to be involved in the progression of PTC, such as the thyroid sarcoidosis, epigenetic changes, environmental exposure, and radiation exposure [3, 4]. PTC patients with certain clinicopathological features have been associated with a poorer prognosis, such as the elder age, larger tumor size, lymph node, or distant metastasis [5–9]. However, the molecular mechanisms remain poorly understood. Therefore, in-depth study of the molecular mechanism involved in the initiation and development of PTC is very important.

One of the molecules of interest in terms of elucidating the mechanism of cancer is microRNAs (miRNAs) family. miRNAs are small noncoding RNAs which are highly conserved and degrade the target mRNAs by binding to their 3′-untranslated region (3′-UTR) [10, 11]. Research on miRNAs for the diagnostic and therapeutic probes has been a hot topic [12–14]. Recent studies implied that miRNAs might serve as new biomarkers for PTC. For example, miRNA-299-5p regulates estrogen receptor alpha and inhibits migration and invasion of papillary thyroid cancer cells [15]. Downregulation of miR-338-3p inhibits PTC progression by repressing AKT3 expression [16].

It has been previously demonstrated that miR-384 (miR-384-3p) exerted the tumor-suppressing role in breast cancer, colorectal cancer, and pancreatic cancer by affecting Wnt, Ras, or AKT pathway [17–20]. However, the specific function and the mechanism of miR-384 in PTC progression require further investigation. The current study aims at delineating the biological function and mechanism of miR-384 in PTC progression and trying to explore novel potential therapeutic target for PTC.

## 2. Materials and Methods

### 2.1. Clinical Samples and Cell Culture

A total of 58 pairs of PTC samples and their paired adjacent noncancerous were obtained from the Pathology Department, Third Affiliated Hospital of Xinxiang Medical University (Xinxiang, China), during the period of January 2017 to June 2018. All the samples were taken directly from intraoperative procedures and then frozen in liquid nitrogen for later use. All the cases had no chemotherapy, radiotherapy, and immunotherapy history. The samples had been diagnosed and divided into PTC and adjacent noncancerous by two independent pathologists who were blinded to the clinical results on the basis of hematoxylin-eosin (HE) staining. The medical records including the age, gender, tumor size, and lymph node metastasis of the patients were collected. The study had been approved by the Ethics Committee of Xinxiang Medical University (Xinxiang, China).

Human PTC cell lines of BCPAP and K1 purchased from American Type Culture Collection (ATCC) were cultured in RPMI-1640 (Invitrogen) supplemented with 10% fetal bovine serum (FBS, Gibco) and 1% penicillin/streptomycin (Invitrogen). The cells were cultured in a humidified incubator with 5% CO_2_ at 37°C.

### 2.2. RNA Extraction and Quantitative Real-Time PCR

Total RNA was isolated from the fresh PTC, adjacent noncancerous tissues, and the cultured PTC cells with TRIzol (Invitrogen, USA) according to the manufacturer's instruction. 2 *μ*g of total RNA was reverse-transcribed to cDNA, and the quantitative detection of miR-384 was performed via the All-in-One TM miRNA real-time PCR Detection Kit (GeneCopoeia, China) by the Applied Biosystems 7500 Sequence Detection system as previously described [16]. U6 or GAPDH was used as internal control. The data were calculated with the 2^–ΔΔCT^ method. The primers were supplied in Supplemental Tables.

### 2.3. Western Blot

The concentration of protein lysates extracted from the PTC cells was detected by BCA Protein Assay Reagent (Thermo Scientific, USA). To separate the protein, the protein lysates were subject to 10% sodium dodecyl sulfate-polyacrylamide gel electrophoresis (SDS-PAGE). Then the separated protein was transferred onto polyvinylidene difluoride (PVDF, Merck Millipore) membranes. Next, the 5% nonfat dry milk was used to block the PVDF membranes at room temperature. The PVDF membranes were cultured with the primary antibodies of anti-PRKACB (rabbit, 1 : 300, Proteintech, USA), anti-ERK1/2 (CST, USA), anti-CREB (rabbit, 1 : 500, CST, USA), anti-p-ERK1/2 (Thr202/Tyr204) (CST, USA) and anti-p-CREB (Ser133) (rabbit, 1 : 500, CST, USA), anti-a-tubulin (mouse, 1 : 2000, Proteintech, USA), and the appropriate HRP-conjugated secondary antibodies (1 : 5000, CST, USA). At last, the specific protein bands on the membranes were measured by chemiluminescence imaging analysis system (Tanon, China).

### 2.4. MTT Assay, Soft Agar Assay, Transwell Migration Assay, Wound-Healing Assay, and Immunohistochemistry

The details of MTT assay, soft agar assay, Transwell migration assay, wound-healing assay, and immunohistochemistry (IHC) are shown in the supplementary materials and methods (Supplementary Materials and Methods).

### 2.5. Plasmid Construction, Transfection, and Dual-Luciferase Reporter Assays

The full length of the 3′-UTR PRKACB gene is 3209 bp, and the binding site of miR-384 was located at 4421–4428 bp. Then, we PCR-amplified the 3′-UTR region of 4321–4490 bp inverted it into the psiCHECK-2 luciferase reporter plasmid (Promega, China) at the site of XhoI/NotI. Cells were seeded on 24-well plates (1 × 10^5^/well) and cultured in 5% CO_2_ at 37°C. The next day, the cells were cotransfected with the mir-384 mimic, psiCHECK-2-luciferase reporter gene plasmids psiCHECK-2-PRKACB-3′-UTR, or their control plasmids using the Lipofectamine 2000 Reagent (Invitrogen, USA) following the manufacturer's protocol. 48 hours later after the transfection, luciferase and renilla activities were detected by Dual-Luciferase Reporter Assay Kit (Promega, China) according to the manufacturer's instructions. Each experiment was performed in triplicate.

### 2.6. Tumorigenesis in Nude Mice

The animal experiments were performed on 4-to 6-week-old BABL/c nude mice which were obtained from the Center of Laboratory Animal Science of Guangdong (Guangzhou, China) according to the Chinese regulations and standards for using laboratory animals. 2 × 10^6^ cells of K1/miR-384, K1/miR-384, and K1/miR-384 + PRKACB were injected subcutaneously in the hind limbs (*n* = 4 for each group). Then, a slide caliper was used to measure the tumor size every 6 days (volume = length × width × height). 3 weeks later, the mice were euthanized and the tumors were excised. The tumors were fixed in 4% paraformaldehyde and embedded in paraffin, and 4 *μ*m sections were prepared and stained with HE or IHC. The primary antibody of Ki-67 was purchased from Maixin (Fuzhou, China).

### 2.7. Statistical Analysis

The statistical analyses were performed with SPSS20.0 for Windows. Data of the study were shown as the means ± standard deviations (mean ± SD). Student's *t*-test or one-way ANOVA with post hoc contrasts by LSD test was conducted to compare the means. *p* < 0.05 was considered as statistically significant. Mann–Whitney *U*-test was performed to compare the medians. The relationship between miR-384 expression and PRKACB mRNA expression was analyzed by Spearman's correlation analysis. *p* values  < 0.05 are indicated by *∗*, and *p* values  < 0.01 are indicated by *∗∗*.

## 3. Results

### 3.1. miR-384 Expression Was Decreased and Correlated with the Clinicopathological Characteristics in PTC

**Figure 1:**
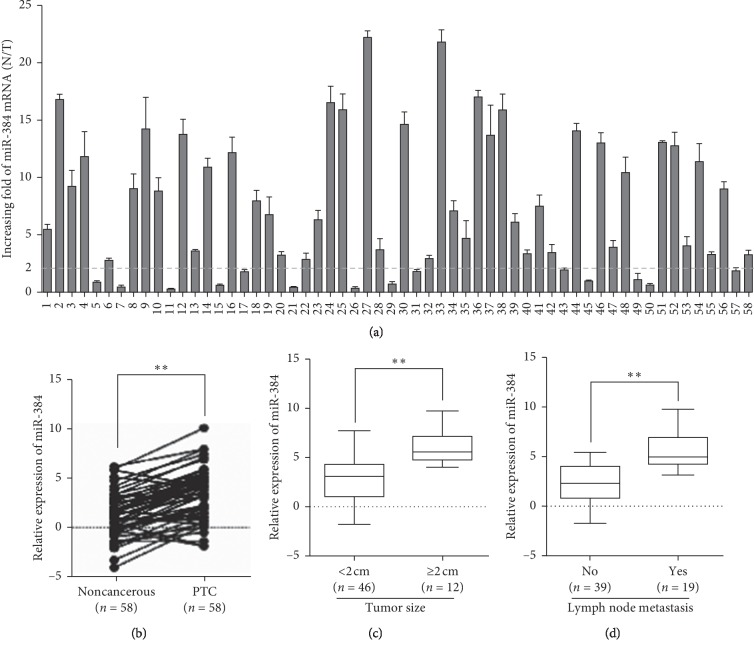
miR-384 was decreased and correlated with the clinicopathological characteristics of PTC. (a) Expression of miR-384 in 58 cases of fresh human PTC tissues and their paired adjacent noncancerous tissues by real-time PCR analysis; miR-384 expression was normalized to U6 and expressed relative to the matched adjacent normal tissues (2^−ΔΔCт^). (b) Mean expression of miR-384 in 58 cases of fresh human PTC tissues and their paired adjacent noncancerous tissues by real-time PCR (ΔCт, mean ± SD, *n* = 58, ^*∗∗*^*p* < 0.01). (c) Expression of miR-384 by real-time PCR according to the primary tumor size (ΔCт, mean ± SD, *n* = 58, ^*∗∗*^*p* < 0.01). (d) Expression of miR-384 by real-time PCR according to the lymph node metastasis (ΔCт, mean ± SD, *n* = 58, ^*∗∗*^*p* < 0.01).

The KM Plotter analysis revealed that the low miR-384 expression showed poor prognosis in thyroid carcinoma patients (*p*=0.0007) ([Supplementary-material supplementary-material-1]). To further explore the expression and significance of miR-384 in PTC, real-time PCR was firstly used to investigate miR-384 expression in 58 cases of fresh PTC and their paired adjacent noncancerous tissues. The results demonstrated that the expression of miR-384 was reduced in 83.0% (49/58) of PTC (T) compared with their paired adjacent noncancerous tissues (N). And among them, the twofold difference (N/T > twofold) was shown in 44 cases (75.0%) (Figure 1(a)). Student's *t*-test revealed that miR-384 expression reduced obviously in PTC (Figure 1(b)). In addition, we analyzed the correlation between miR-384 expression and clinicopathological characters. The results of Mann–Whitney *U*-test demonstrated the expression was much higher in tumors with lymph node metastasis, in larger size than that without lymph node metastasis, in smaller size (Figures 1(c) and 1(d) and Table 1). The above results showed that the reduced expression of mir-384 might play a crucial role during the PTC progression.

### 3.2. Ectopic Overexpression of miR-384 Suppressed PTC Progression

**Figure 2:**
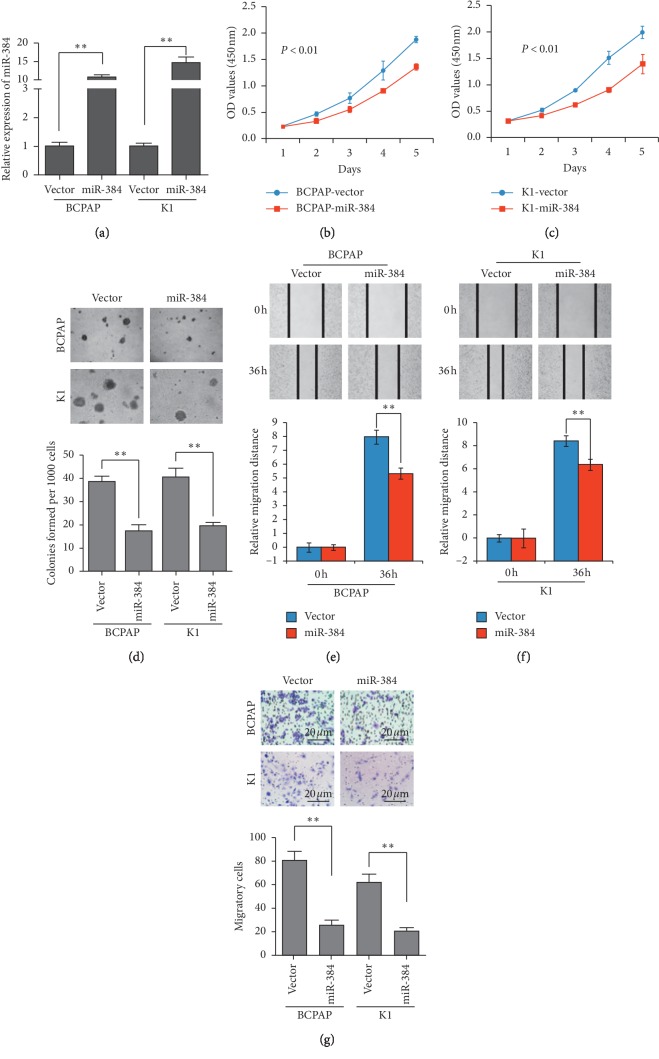
Overexpression of miR-384 inhibited the progression of PTC cells. (a) Overexpression of miR-384 in BCPAP and K1 cells verified by real-time PCR. (b–d) The proliferative ability of the indicated cells detected by MTT assays and soft agar assays. Only cell colonies containing more than 50 cells were counted. Error bars represent mean ± SD from 3 independent experiments. (e–f) Representative images of wound-healing assay (original magnification, ×100). Histograms represent the average migrated distances at the indicated times. Error bars represent mean ± SD from three independent experiments. (g) Transwell migration assay. Representative images (left) and quantification (right) of migrated cells across a Transwell chamber. ^*∗∗*^*p* < 0.01.

To investigate the role of miR-384 in PTC progression, hsa-miR-384 mimics were transfected into BCPAP and K1 cells and the cells with ectopic overexpression of miR-384 were obtained (Figure 2(a)). MTT, soft agar assay, and wound-healing assay were conducted to explore the function of miR-384 on the PTC progression. The results of MTT and soft agar assay revealed that the proliferation of PTC reduced significantly by the ectopic overexpression of miR-384 (Figures 2(b)–2(d)). The result of Transwell migration assay and wound-healing assay showed that the migrated ability of PTC cells was obviously suppressed by the ectopic overexpression of miR-384 (Figures 2(e) and 2(f)). Therefore, the ectopic overexpression of miR-384 suppressed PTC progression.

### 3.3. Suppression of Endogenous miR-384 Promoted PTC Progression

**Figure 3:**
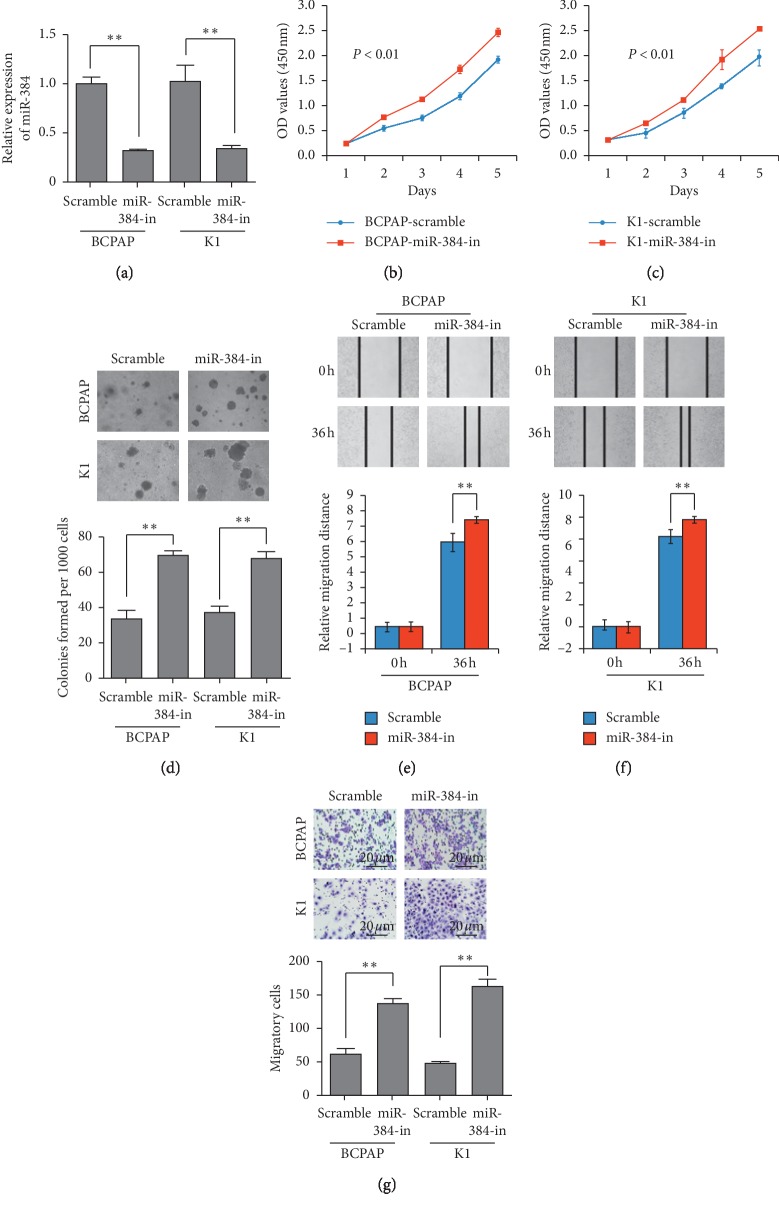
Inhibition of endogenous miR-384 promoted the progression of PTC cells. (a) Expression of miR-384 in BCPAP and K1 cells transfected with inhibitor or their paired negative control lentiviral vector by real-time PCR. (b–d) The proliferative ability of the indicated cells detected by MTT assays and soft agar assays. Only cell colonies containing more than 50 cells were counted. Error bars represent mean ± SD from 3 independent experiments. (e–f) Representative images of wound-healing assay (original magnification, ×100). Histograms represent the average migrated distances at the indicated times. Error bars represent mean ± SD from three independent experiments. (g) Transwell migration assay. Representative images (left) and quantification (right) of migrated cells across a Transwell chamber. ^*∗∗*^*p* < 0.01.

Then, we transfected miR-384 inhibitors into BCPAP and K1 cells and obtained the cells with endogenous suppression of miR-384 (Figure 3(a)). Results of MTT assay, soft agar assay, Transwell migration assay, and wound-healing assay demonstrated that suppression of miR-384 could significantly promote the proliferative and migratory abilities of the BCPAP and K1 cells compared with their negative control cells (Figures 3(b)–3(f)). So, miR-384 suppression promoted the progression of PTC.

### 3.4. miR-384 Directly Targeted the 3′-UTR of PRKACB Gene

**Figure 4:**
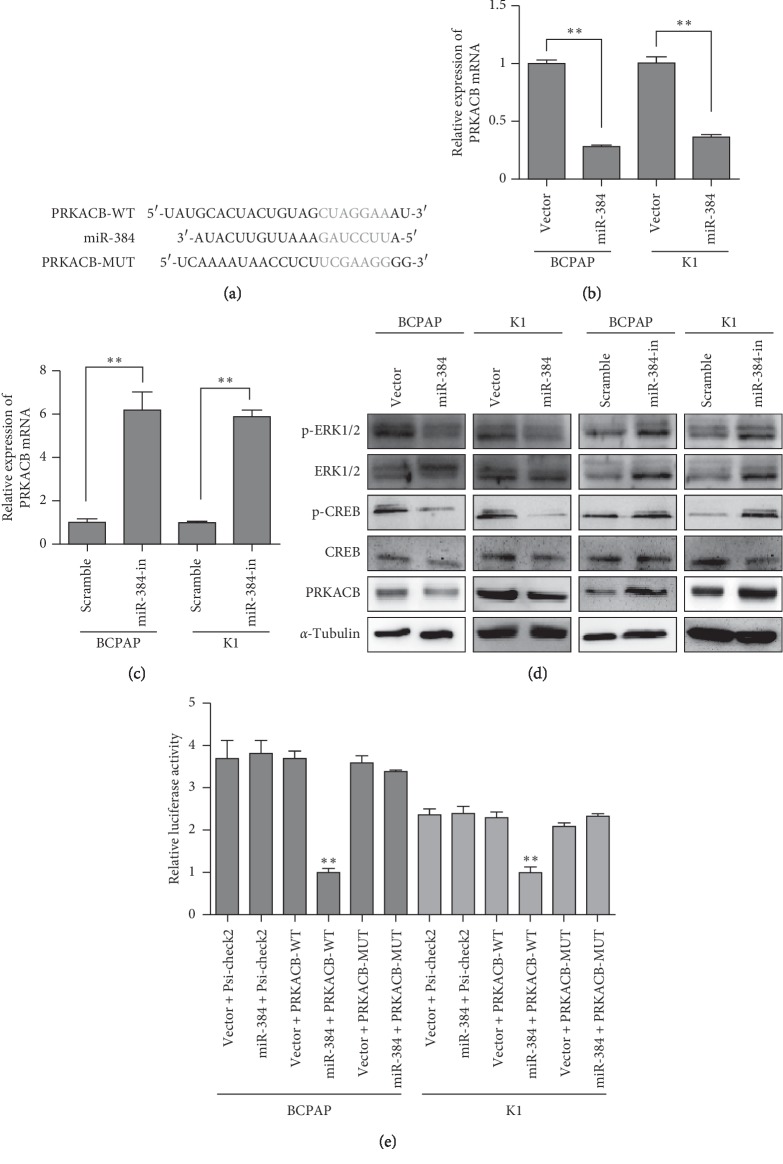
miR-384 decreased PRKACB expression by directly binding to its 3′-UTR. (a) Predicted miR-384 target sequences in the 3′-UTRs of PRKACB and their mutants containing altered nucleotides in the 3′-UTRs. (b–c) Real-time PCR analysis of PRKACB. (d) Western blot analysis of PRKACB as well as the activation of PKA downstream effectors (in the indicated cells). (e) Luciferase assay analyses of the indicated cells transfected with the indicated reporters with increasing amounts of miR-384 (Error bars represent mean ± SD from three independent experiments; *p* < 0.01).

The publicly available bioinformatics algorithms (TargetScan and miRDB) were used to predict the theoretical target gene of miR-384. And PRKACB gene was found to be a potential target of miR-384 (Figure 4(a)). To observe the response of PRKACB toward miR-384, real-time PCR and western blot were used to determine the expression of PRKACB mRNA and protein. It was found that both PRKACB mRNA and the protein were obviously decreased with the overexpression of miR-384, and miR-384 suppression had the opposite effects (Figures 4(b)–4(d)). In addition, we detected the activation of PKA downstream effectors of p-ERK1/2 and P-CREB. It was found that they were also suppressed by miR-384. To further confirm the direct interaction between miR-384 and PRKACB, the dual-luciferase reporter assay system was conducted. As shown in Figure 4(e), the luciferase activity was remarkably suppressed when cells were cotransfected with miR-384 mimic and wild-type PRKACB 3′-UTR. But no obvious differences were observed when cotransfected with the mutant 3′-UTR constructs or their scramble vectors. The above results verified that PRKACB was the target gene of miR-384. Collectively, these results indicated that miR-384 regulated the expression of PRKACB gene by directly binding its 3′-UTR. Our results provided evidence on the direct inhibiting role of miR-384 on the PRKACB, thus attenuating the PKA activity.

### 3.5. miR-384 Suppressed PTC Progression through Inhibition of PRKACB

**Figure 5:**
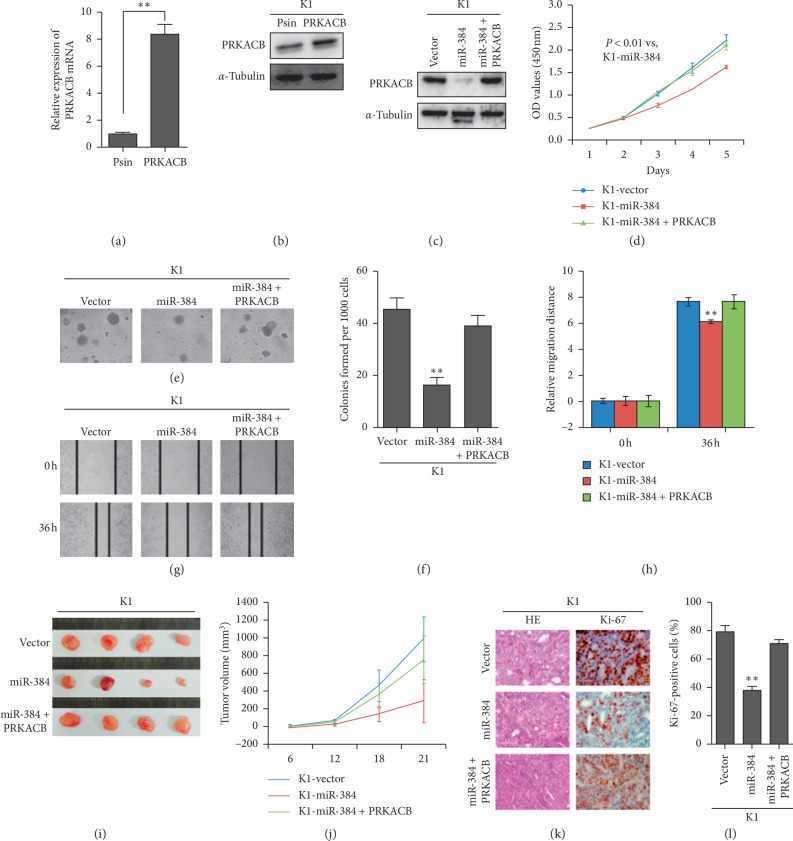
miR-384 inhibited the progression of PTC cells by targeting PRKACB. (a–c) PRKACB overexpression in K1 cells by real-time PCR analysis and western blot. (d–f) The proliferative ability of the indicated cells detected by MTT assays and soft agar assays. Only cell colonies containing more than 50 cells were counted. Error bars represent mean ± SD from 3 independent experiments. (g, h) Representative images of wound-healing assay (original magnification, ×100). Histograms represent the average migrated distances at the indicated times. Error bars represent mean ± SD from three independent experiments. (i, j) K1/miR-384, K1/miR-384 + PRKACB, and K1/Vector cells were injected into the hind limbs of nude mice (*n* = 4). Tumor volumes were measured on the indicated days. The tumor volume data were presented as the mean ± SD. (k, l) Histopathological analyses of xenograft tumors. The tumor sections were stained with HE or subjected to IHC staining using an antibody against Ki-67. Error bars represent mean ± SD from three independent experiments. ^*∗∗*^*p* < 0.01.

To further understand whether PRKACB gene was involved in miR-384-mediated PTC progression, the expression of PRKACB gene was restored in K1/miR-384 and BCPAP/miR-384 cells (Figures 5(a)–5(c) and Figures [Supplementary-material supplementary-material-1]–[Supplementary-material supplementary-material-1]) by transfecting the PRKACB gene ORF constructs without 3′-UTRs. Then, we conducted the MTT assay, soft agar assay, and wound-healing assay in the vector cells and miR-384-overexpressing cells, and the miR-384-overexpressing cells restored PRKACB gene. The results verified that the ectopic overexpression of PRKACB gene could reverse the influence of miR-384 on PTC proliferation and migration (Figures 5(d)–5(h) and [Supplementary-material supplementary-material-1]–[Supplementary-material supplementary-material-1]). To further observe the in vivo effects of miR-384 in PTC cells, we performed the tumorigenesis assay in nude mice with K1/Vector, K1/miR-384, and K1/miR-384 + PRKACB cells. It was found that the tumor size in K1/miR-384 group was smaller than that in K1/Vector group (Figures 5(i) and 5(j)). However, when we restored the expression of PRKACB gene in K1/miR-384 cells, the tumor size increased (Figures 5(i) and 5(j)). The results of IHC demonstrated that the tumors of K1/miR-384 group had lower Ki-67 indices than that in the Vector group (Figures 5(k) and 5(l)). However, when we restored the expression of PRKACB gene in K1/miR-384cells, the Ki-67 indices increased (Figures 5(k) and 5(l)).

### 3.6. Correlation between miR-384 and PRKACB Expression

**Figure 6:**
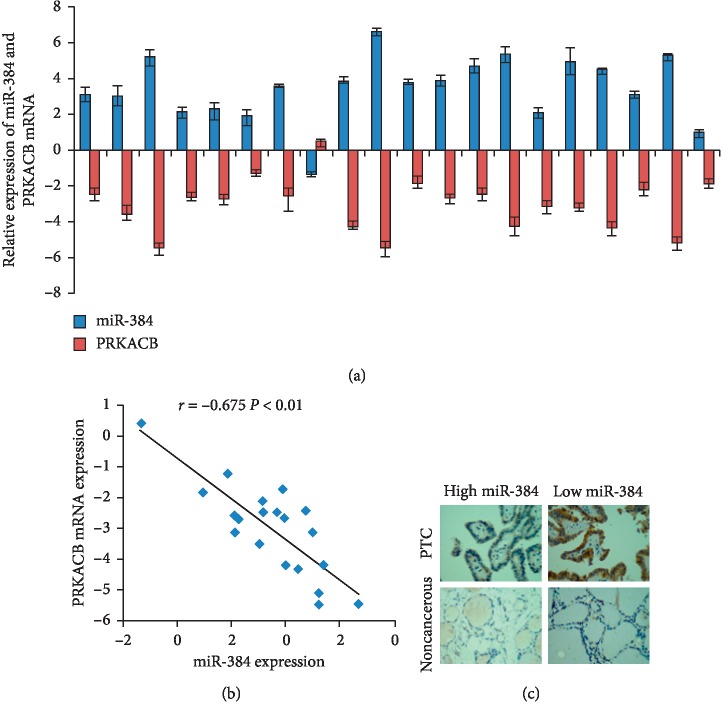
Clinical data confirmed PRKACB was the direct target of miR-384. (a) The expression of miR-384 and PRKACB mRNA was detected by real-time PCR (ΔCт, *n* = 20). (b) Spearman's correlation analysis of miR-384 expression and PRKACB mRNA expression. (c) IHC detection of PRKACB protein in PTC and their paired noncancerous samples.

To further explore whether the above results could be supported by clinical tissues, we analyzed the expression of miR-384 and PRKACB mRNA in the same 20 cases of fresh PTC tissues by real-time PCR. The results showed that the expression level of PRKACB mRNA was higher in the cases with lower miR-384 expression than that with higher miR-384 expression (Figure 6(a)). And the Spearman correlation analyses revealed that there was a negative correlation between PRKACB mRNA and miR-384 expression (Figure 6(b)). In addition, the expression of PRKACB protein in the 58 cases of PTC and their paired noncancerous tissues was detected by IHC. The results of IHC showed that the expression of PRKACB protein was mainly localized in the cytoplasm (Figure 6(c)). The IRS analysis revealed that PRKACB protein was highly expressed in 47 (81.0%) cases of PTC samples and 8 (13.8%) cases of noncancerous samples. Further analysis revealed that PRKACB protein was highly expressed in 45 cases of PTC samples with low miR-384 expression (49 cases), while it was lowly expressed in 4 cases of PTC samples in those with high miR-384 expression (9 cases). These findings were in accordance with the results of qPCR.

## 4. Discussion

It is well known that thyroid carcinoma (TC), especially PTC, is the most common type of endocrine malignancy [21]. As we know, the prognosis of PTC patients is much better than most of the other malignant tumors. It has been reported that the 5-year survival rate of PTC was more than 95% [22]. However, some of them might develop into more aggressive thyroid cancers. Moreover, the recurrence was found in about 30% of the PTC patients [23]. So, it is necessary to further explore the molecular characteristics of PTC.

MicroRNAs are small noncoding RNAs which could negatively regulate the expression of the target genes by directly binding their 3′-UTRs [24]. It has been found that the deregulation of miRNA expression is a common feature of many types of human cancers, including thyroid cancer [25, 26]. Accumulated evidence has demonstrated that the aberrant expression of miRNAs plays crucial roles in cancer initiation and progression [27–30]. In this article, we explored the relationship between the dysregulated miR-384 and the progression of PTC. We detected miR-384 expression in 58 cases of PTC and their matched adjacent noncancerous tissues by real-time PCR. The results showed that miR-384 expression remarkably decreased in PTC, especially in cases with lymph node metastasis, elder patients, and female patients. Furthermore, the expression level of miR-384 was obviously lower in the cases with larger tumor size than those with smaller size. In addition, we found that the proliferation and migration of PTC cells were obviously suppressed by miR-384 overexpression. Moreover, the suppression of miR-384 remarkably increased the proliferation and migration of PTC. The above results agreed with the previous study of Sun et al. [31]. In brief, miR-384 plays a role of cancer suppressor. Therefore, it is necessary to further explore the underlying mechanism of miR-384 in suppressing PTC progression.

It is well known that miRNAs modulate their target gene expression by partially pairing with the 3′-UTRs and about two-thirds of human mRNAs were regulated by miRNAs [32]. In the current study, cAMP-dependent protein kinase catalytic beta subunit (PRKACB) was selected as the theoretical target gene of miR-384 by the analysis of prediction software. PRKACB gene has been identified to be an important oncogene in cancer progression, especially in the progression of endocrine cancers by modulating cAMP signaling activity [33, 34]. Moreover, it was recently found that miR-302a-3p suppresses hepatocellular carcinoma progression by targeting the 3′-UTR of PRKACB gene [35].

We next conducted dual-luciferase reporter assay, real-time RT-PCR, and western blot to further explore whether PRKACB was exactly the target gene of miR-384 in PTC. Our results provided evidence on the direct inhibiting role of miR-384 on the PRKACB, thus attenuating the PKA activity. It is thus reasonable that miR-384 may suppress PTC progression at least partially by impairing PKA signal transduction pathway. In addition, it was found that the inhibition role of miR-384 in PTC progression could be rescued by the overexpression of PRKACB gene. Furthermore, it was found that PRKACB gene expression increased obviously in human PTC tissues compared with their paired noncancerous samples. In addition, there was a negative correlation between miR-384 and PRKACB gene expression. All of the results verified that miR-384 suppressed PTC progression by directly targeting PRKACB gene.

In summary, our study confirmed that the downregulation of miR-384 is an independent prognostic factor for poorer prognosis of PTC patients, and miR-384 inhibits PTC progression by directly targeting PRKACB gene. Therefore, miR-384/PRKACB might be a novel potential therapeutic target for PTC.

## Figures and Tables

**Table 1:** Clinicopathologic characteristics of miR-384 expression in PTC patients.

Clinicopathological variables	miR-384 expression		*p* value
	Low	High	
Age (years)			
<45	6	12	0.089
≥45	23	17	
Gender			
Male	7	13	0.097
Female	22	16	
Tumor size (cm)			
<2	17	27	0.002
≥2	12	2	
N classification			0.000
Yes	17	2	
No	12	27	

## Data Availability

The materials and the relevant raw data will be freely available to any scientist wishing to use them for noncommercial purposes from the corresponding author.

## Supplementary Materials

Supplementary Materials Table S1: primer sequences used for target gene real-time PCR. Table S2: primer sequences used for amplification and plasmid construction. Table S3: sequences of miR-384 mimic and inhibitor. Figure S1: KM plotter analysis of miR-384 in thyroid carcinoma. Figure S2: miR-384 inhibited the progression of PTC cells by targeting PRKACB.

## References

[1] Sondermann A., Andreghetto F. M., Moulatlet A. C. B. (2015). MiR-9 and miR-21 as prognostic biomarkers for recurrence in papillary thyroid cancer. *Clinical & Experimental Metastasis*.

[2] Farahati J., Mäder U., Gilman E. (2019). Changing trends of incidence and prognosis of thyroid carcinoma. *Nuklearmedizin*.

[3] Zheng H., Wang M., Jiang L. (2016). BRAF-activated long noncoding RNA modulates papillary thyroid carcinoma cell proliferation through regulating thyroid stimulating hormone receptor. *Cancer Research and Treatment*.

[4] Schneider A. B., Sarne D. H. (2005). Long-term risks for thyroid cancer and other neoplasms after exposure to radiation. *Nature Clinical Practice Endocrinology & Metabolism*.

[5] Deng X., Wu B., Xiao K. (2015). MiR-146b-5p promotes metastasis and induces epithelial-mesenchymal transition in thyroid cancer by targeting ZNRF3. *Cellular Physiology and Biochemistry*.

[6] Shi R., Qu N., Liao T., Wei W.-J., Wang Y.-L., Ji Q.-H. (2016). The trend of age-group effect on prognosis in differentiated thyroid cancer. *Scientific Reports*.

[7] Yin D. T., Yu K., Lu R. Q., Li X., Xu J., Lei M. (2016). Prognostic impact of minimal extra-thyroidal extension in papillary thyroid carcinoma. *Medicine*.

[8] Czarniecka A., Kowal M., Rusinek D. (2015). The risk of relapse in papillary thyroid cancer (PTC) in the context of BRAF^V600E^ mutation status and other prognostic factors. *PLoS One*.

[9] Adam M. A., Pura J., Goffredo P. (2015). Presence and number of lymph node metastases are associated with compromised survival for patients younger than age 45 years with papillary thyroid cancer. *Journal of Clinical Oncology*.

[10] Paulmurugan R. (2013). MicroRNAs—a new generation molecular targets for treating cellular diseases. *Theranostics*.

[11] Zhang G. J., Li J. S., Zhou H., Xiao H.-X., Li Y., Zhou T. (2015). MicroRNA-106b promotes colorectal cancer cell migration and invasion by directly targeting DLC1. *Journal of Experimental & Clinical Cancer Research*.

[12] Kloosterman W. P., Plasterk R. H. A. (2006). The diverse functions of MicroRNAs in animal development and disease. *Developmental Cell*.

[13] He L., Hannon G. J. (2004). MicroRNAs: small RNAs with a big role in gene regulation. *Nature Reviews Genetics*.

[14] Bonfrate L., Altomare D. F., Lena M. (2013). MicroRNA in colorectal cancer: new perspectives for diagnosis, prognosis and treatment. *Journal of Gastrointestinal and Liver Diseases*.

[15] Wang Z., He L., Sun W. (2018). miRNA-299-5p regulates estrogen receptor alpha and inhibits migration and invasion of papillary thyroid cancer cell. *Cancer Management and Research*.

[16] Sui G. Q., Fei D., Guo F. (2017). MicroRNA-338-3p inhibits thyroid cancer progression through targeting AKT3. *American Journal of Cancer Research*.

[17] Wang Y., Zhang Z., Wang J. (2018). MicroRNA-384 inhibits the progression of breast cancer by targeting ACVR1. *Oncology Reports*.

[18] Wang Y. X., Chen Y. R., Liu S. S. (2016). MiR-384 inhibits human colorectal cancer metastasis by targeting KRAS and CDC42. *Oncotarget*.

[19] Wang Y. X., Zhu H. F., Zhang Z. Y., Ren F., Hu Y.-H. (2018). MiR-384 inhibits the proliferation of colorectal cancer by targeting AKT3. *Cancer Cell International*.

[20] Wang G., Pan J., Zhang L., Wei Y., Wang C. (2017). Long non-coding RNA CRNDE sponges miR-384 to promote proliferation and metastasis of pancreatic cancer cells through upregulating IRS1. *Cell Proliferation*.

[21] Nguyen Q. T., Lee E. J., Huang M. G., Park Y. I, Khullar A, Plodkowski R. A (2015). Diagnosis and treatment of patients with thyroid cancer. *American Health and Drug Benefits*.

[22] Bray F., Ferlay J., Soerjomataram I., Siegel R. L., Torre L. A., Jemal A. (2018). Global cancer statistics 2018: GLOBOCAN estimates of incidence and mortality worldwide for 36 cancers in 185 countries. *CA: A Cancer Journal for Clinicians*.

[23] Xing M., Alzahrani A. S., Carson K. A. (2015). Association between BRAF V600E mutation and recurrence of papillary thyroid cancer. *Journal of Clinical Oncology*.

[24] Ambros V. (2004). The functions of animal microRNAs. *Nature*.

[25] Liu C., Feng Z., Chen T. (2019). Downregulation of NEAT1 reverses the radioactive iodine resistance of papillary thyroid carcinoma cell via miR-101-3p/FN1/PI3K-AKT signaling pathway. *Cell Cycle*.

[26] Jiang W., Zhan H., Jiao Y., Li S., Gao W. (2018). A novel lncRNA-miRNA-mRNA network analysis identified the hub lncRNA RP11-159F24.1 in the pathogenesis of papillary thyroid cancer. *Cancer Medicine*.

[27] Iorio M. V., Croce C. M. (2017). Micro RNA dysregulation in cancer: diagnostics, monitoring and therapeutics: a comprehensive review. *EMBO Molecular Medicine*.

[28] Pencheva N., Tavazoie S. F. (2013). Control of metastatic progression by microRNA regulatory networks. *Nature Cell Biology*.

[29] Pencheva N., Tran H., Buss C. (2012). Convergent multi-miRNA targeting of ApoE drives LRP1/LRP8-dependent melanoma metastasis and angiogenesis. *Cell*.

[30] Mingozzi F., High K. A. (2011). Therapeutic in vivo gene transfer for genetic disease using AAV: progress and challenges. *Nature Reviews Genetics*.

[31] Sun H., He L., Ma L. (2017). LncRNA CRNDE promotes cell proliferation, invasion and migration by competitively binding miR-384 in papillary thyroid cancer. *Oncotarget*.

[32] Griffiths-Jones S., Saini H. K., van Dongen S., Enright A. J. (2008). miRBase: tools for microRNA genomics. *Nucleic Acids Research*.

[33] Espiard S., Knape M. J., Bathon K. (2018). Activating PRKACB somatic mutation in cortisol-producing adenomas. *JCI Insight*.

[34] Orchel J., Witek L., Kimsa M. (2012). Expression patterns of kinin-dependent genes in endometrial cancer. *International Journal of Gynecologic Cancer*.

[35] Ye Y., Song Y., Zhuang J. (2018). MicroRNA-302a-3p suppresses hepatocellular carcinoma progression by inhibiting proliferation and invasion. *OncoTargets and Therapy*.

