# Supplementary material for: MicroRNA-384 Inhibits the Progression of Papillary Thyroid Cancer by Targeting PRKACB
Source: Biomed Res Int. 2020 Jan 8;2020:4983420. doi: 10.1155/2020/4983420 (PMC6973191; doi:10.1155/2020/4983420)
Supplement: Supplementary Materials — Table S1: primer sequences used for target gene real-time PCR. Table S2: primer sequences used for amplification and plasmid construction. Table S3: sequences of miR-384 mimic and inhibitor. Figure S1: KM plotter analysis of miR-384 in thyroid carcinoma. Figure S2: miR-384 inhibited the progression of PTC cells by targeting PRKACB. [file 4983420.f1.doc]

**Supplementary Tables**

**Table S1.** Primer sequences used for target gene real-time PCR

| **Gene** | **Forward primer** | **Reverse primer** |
| --- | --- | --- |
| **PRKACB** | GATGCATCTGCTTGCTCCT | ACTGTTCAGTGGCTTTGTGT |
| **GAPDH** | GACTCATGACCACAGTCCATGC | AGAGGCAGGGATGATGTTCTG |

**Table S2.** Primer sequences used for amplification and plasmid construction

| **Gene** | **Forward primer** | **Reverse primer** |
| --- | --- | --- |
| **PRKACB-3’UTR-WT** | CCGCTCGAGCCTTGTAAACTGTAACCTAT | GGGGCGGCCGCCCATTGAAGGTACTTTAATT |

**Table S3.** Sequences of miR-384 mimic and inhibitor

| **Gene** | **Sequence** |
| --- | --- |
| **miR-384 mimic** | AUUCCUAGAAAUUGUUCAUA UGAACAAUUUCUAGGAAUUU |
| **miR-384 inhibitor** | UAUGAACAAUUUCUAGGAAU |

**Supplementary Figures:**

**Figure S1**

**
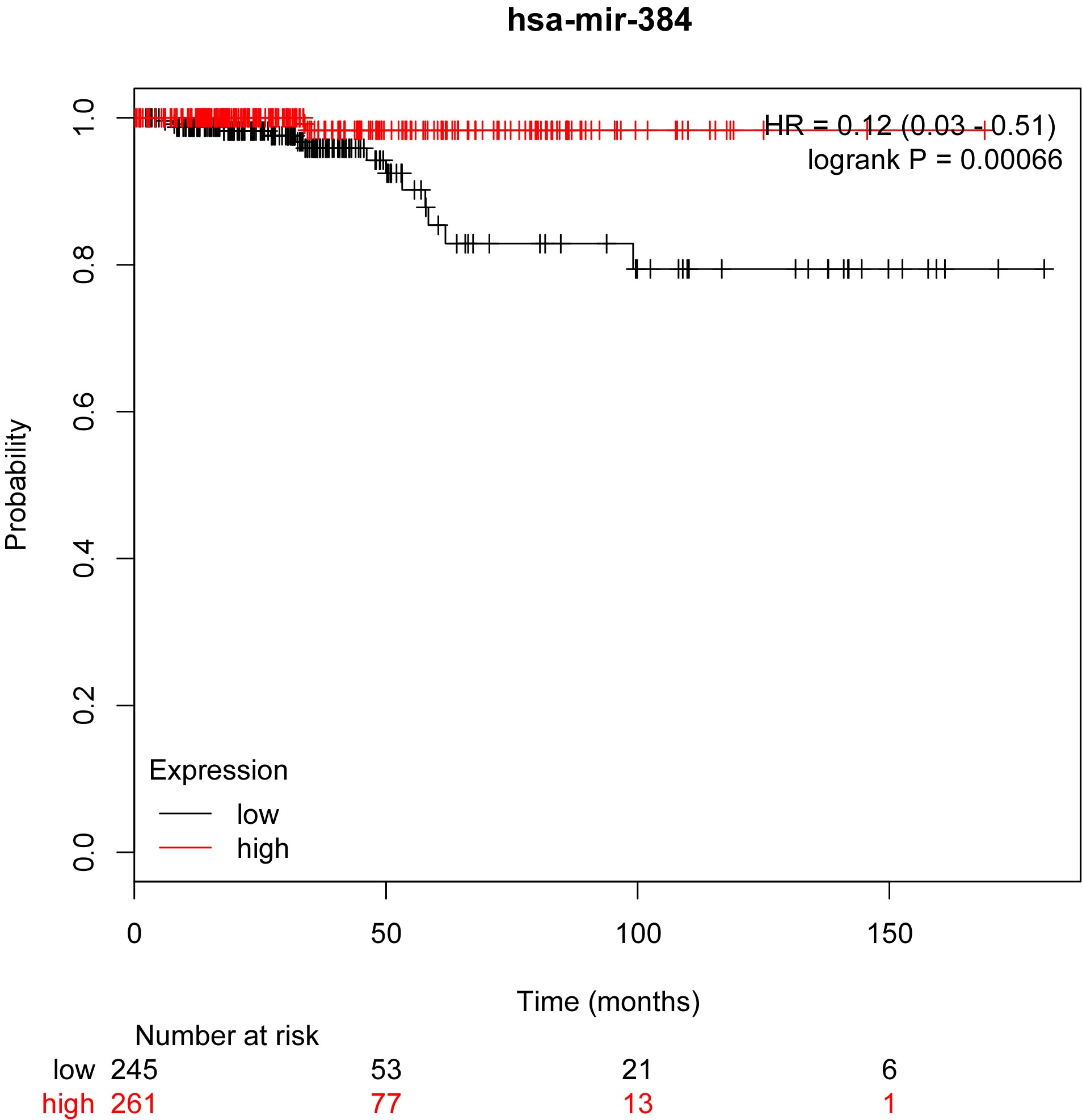
**

**Figure S2**

**
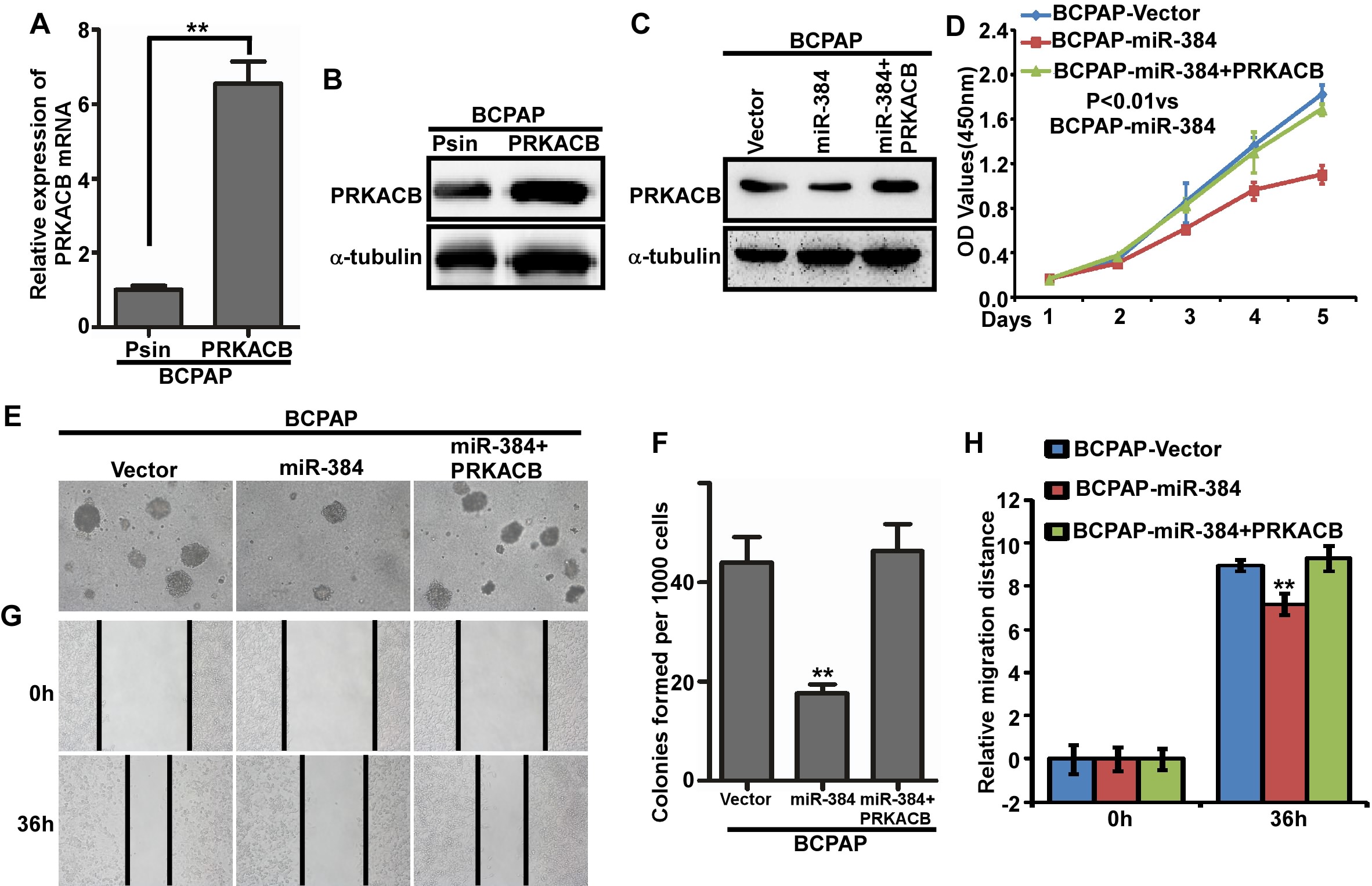
**

**Supplementary Figure legends:**

Figure S1. KM Plotter analysis of miR-384 in thyroid carcinoma.

Figure S2. MiR-384 inhibited the progression of PTC cells by targeting PRKACB. (A-C) PRKACB over-expression in BCPAP cells by real-time PCR analysis and Western blot. (D-F) The proliferative ability of the indicated cells detected by MTT assays and soft agar assays. Only cell colonies containing more than 50 cells were counted. Error bars represent mean±SD from 3 independent experiments. (G, H) Representative images of wound-healing assay. Original magnification, ×100. Histograms represent the average migrated distances at the indicated times. Error bars represent mean±s.d. from three independent experiments. **P<0.01.

**Supplementary Materials and Methods**

*MTT assay.* Cells (1000 cells/well) transfected with the corresponding mimic, the inhibitor or their controls were seeded on 96-well plates.After 24 hours , 20 μl 5 g/l 3-(4,5-dimethylthiazol-z-yl)-2,5-diphenyltetrazolium bromide (MTT, Sigma, USA) was mixed into each well and cultured for 4 h at 37°C in 5% CO_2_. Then, each well was added into 150 μl dimethyl sulphoxide (DMSO; Sigma, USA) after removing MTT. The absorbance of each well was tested at a wavelength of 450 nm with a microplate autoreader (Bio-Rad, Hercules, CA, USA). Each experiment was performed in triplicate.

*Soft agar assay.* Six-well plates covered with 0.6% medium-agar (Sigma, USA) supplemented with 20% fetal bovine serum at the the bottom layer were prepared. 1×10^3^ cells transfected with the corresponding mimic, inhibitor or their controls were mixed with the 0.3% medium-agar and seeded on ech well. Then, they were cultured in 5% CO_2_ at 37°C for 2 weeks. Each assay was performed in triplicate. 2 weeks later, colonies containing at least 50 cells were scored and photographed at an original magnification of ×200. Each experiment was performed in triplicate.

*Wound-healing assay.* Cells transfected with corresponding mimic, inhibitor or their controls were seeded on six-well plates and cultured in 5% CO2 at 37°C until 90% confluence. After serum starvation for 24 h, a (yellow) pipette tip was used to create a straight scratch. Then the indicated positions for observation and photograph were selected at the approprite time. Each experiment was performed in triplicate.

*Transwell migration assay* For Transwell migration assay, A Boyden chamber with 8μm-pore filter membrane was used. Briefly, cells (1×10^5^) in culture medium containing free FBS were seeded in the upper chamber, and the culture medium with 20% FBS was added in the lower chamber as a chemoattractant. After incubation for 48 h, the chamber was fixed in 4% paraformaldehyde and stained with Haematoxylin. Cells on the upper side of the filter were removed with cotton swabs. Cells that migrated to the lower side were fixed in 4% paraformaldehyde and stained with Haematoxylin. The migratory cells on the lower surface of the filter were counted. Three independent experiments were performed and the data were presented as the mean ± s.d..

*Immunohistochemistry* Paraffin-embedded specimens were cut into 4-μm sections and baked at 60°C for 2 h. Immunohistochemistry was performed via SP-9000 detection kits which were were purchased from ZSGB-BIO (Beijing, China). The slides were incubated overnight at 4°C with a primary antibody. As the negative control, PBS was used in place of the primary antibody. The sections were incubated with 3,3-diaminobenzidine (DAB) for 1 min and counterstained with Hematoxylin and dehydrated with a gradient alcohol series and xylenes. The slides were sealed with neutral balsam. The sections were evaluated and scored independently by two researchers, who were blind to the patient outcomes, based on both the proportion of positively stained tumor cells and the intensity of the staining.
